# miR-375 Promotes Pancreatic Differentiation *In Vitro* by Affecting Different Target Genes at Different Stages

**DOI:** 10.1155/2021/6642983

**Published:** 2021-04-07

**Authors:** Zhenyu Lu, Jing Wang, Xu Wang, Zhiying Li, Dan Niu, Min Wang, Jinzhu Xiang, Yongli Yue, Yajuan Xia, Xueling Li

**Affiliations:** ^1^State Key Laboratory of Reproductive Regulation and Breeding of Grassland Livestock, School of Life Sciences, Inner Mongolia University, Hohhot, China; ^2^Inner Mongolia Center for Disease Control and Prevention, Hohhot, China

## Abstract

Human embryonic stem cells (hESCs) possess the ability to differentiate into insulin-producing cells (IPCs), which can be used to treat type I diabetes. miR-375 is an essential transcriptional regulator in the development and maturation of the pancreas. In this study, we optimized a protocol to differentiate hESCs into IPCs and successfully obtained IPCs. Then, we performed overexpression and inhibition experiments of miR-375 on cells at different stages of differentiation and performed RNA-seq. The results showed that the expression of miR-375 fluctuated during hESC differentiation and was affected by miR-375 mimics and inhibitors. miR-375 influences global gene expression and the target genes of miR-375. The overexpression of miR-375 can cause changes in multiple signaling pathways during pancreatic development. miR-375 is a major participant in the differentiation of pancreatic *β*-cells through different target genes at different stages. This study provides new ideas for further investigation of how microRNAs affect cell fate and gene transcription.

## 1. Introduction

MicroRNAs (miRNAs) are small noncoding RNAs that usually bind to the 3′-untranslated regions (3′-UTRs) of target genes and generally function as negative regulators of gene transcription [1, 2]. miRNAs control the expression of many genes [3] and are involved in crucial biological processes, including development, differentiation, apoptosis, cell proliferation, and disease [4–8]. The precise control of insulin secretion in *β*-cells is important to maintain blood glucose stability; insulin secretion disorders may lead to chronic hyperglycemia and diabetes [9]. miRNAs play an important role in insulin secretion, as well as pancreatic islet development, *β*-cell differentiation, glucolipid metabolism, and many other diabetes-related processes and complications [10]. The regulatory effects of miR-30 and miR-200 on the epithelial-to-mesenchymal transition may be an effective way to control islet cell development [11]. Compared with the control samples, the expression of 11 miRNAs, including miR-21-5p, miR-24-3p, miR-100-5p, miR-146a-5p, miR-148a-3p, miR-150-5p, miR-181a-5p, miR-210-5p, miR-342-3p, miR-375, and miR-1275, was observed in the type I diabetes mellitus (T1DM) [12]. *Reg1* may be the direct target of miR-7 that causes its effect on pancreatic *β*-cell function [13]. miRNA-224 may be used as an indicator of *β*-cell death [14]. miR-377 and miR-216a are considered early biomarkers of type I diabetic nephropathy in children [15].

miR-375 plays an important role in the complex network regulating pancreatic development, which includes genes such as *NEUROD1*, *NGN3*, *PDX1*, and *HNF6* [16]. In male patients with diabetes caused by excessive nutrition intake, which is characterized by a progressive increase in insulin secretion, *β*-cell death, and miR-375 expression [17], miR-375 expression was correlated with *∆*C-peptide; in turn, this was associated with insulin production and secretion, which is an important marker in the detection of islet function [18]. Meanwhile, miR-375 plays an important role in pancreatic development. It has been reported that miR-375 directly regulates *PDK1* mRNA expression and reduces its protein level, reducing glucose stimulation, which triggers the expression of insulin and DNA synthesis in *β*-cells [19]. Myotrophin (Mtpn) was predicted to be a target of miR-375 [20]. The inhibitory effects of *Mtpn* short-interfering RNA (siRNA) on glucose-stimulated insulin secretion and exocytosis are similar to that of miR-375. miR-375 is transcriptionally inhibited by the cAMP-protein kinase A (PKA) pathway. This inhibition is achieved by blocking the binding of RNA polymerase II to the miR-375 promoter [21]. In addition, miR-375 exerts its function through the regulation of genes related to pancreatic development, cell growth and proliferation, and insulin secretion.

Stem cells have the ability to self-renew and the potential for differentiation in multiple lineages. They can differentiate into different types of cells under different conditions. The differentiation of human embryonic stem cells (hESCs) into insulin-producing cells (IPCs) is one of the hot topics of current research. In recent years, it has been reported that human-induced pluripotent stem cells (hiPSCs) [22] and hESCs [23] have been successfully induced to form *β*-like cells in vitro. However, the differentiated cells have the problems of low insulin secretion and the coexpression of glucagon and somatostatin. miR-375 regulates many important regulators in the differentiation of hESCs to IPCs, such as *PDPK1* [24], *HNF1β* [25], and *NOTCH*2 [26]. Although research into miR-375 and its target genes is relatively comprehensive, it remains necessary to analyze how miR-375 participates in the entire process of ESC differentiation into IPCs.

In this study, we optimized the reported differentiation protocol of hESCs to IPCs and successfully obtained insulin-like cells. miR-375 was overexpressed or inhibited at different stages of differentiation, and the cells were analyzed by RNA-Seq. The effects of miR-375 on global transcriptional changes during pancreatic differentiation in vitro and the network of pancreas-specific transcription factors and miR-375 target genes have been illustrated. In addition, we attempted to determine how miR-375 affected the pancreatic differentiation and insulin production in hESCs.

## 2. Materials and Methods

### 2.1. Cell Culture

**Figure 1:**
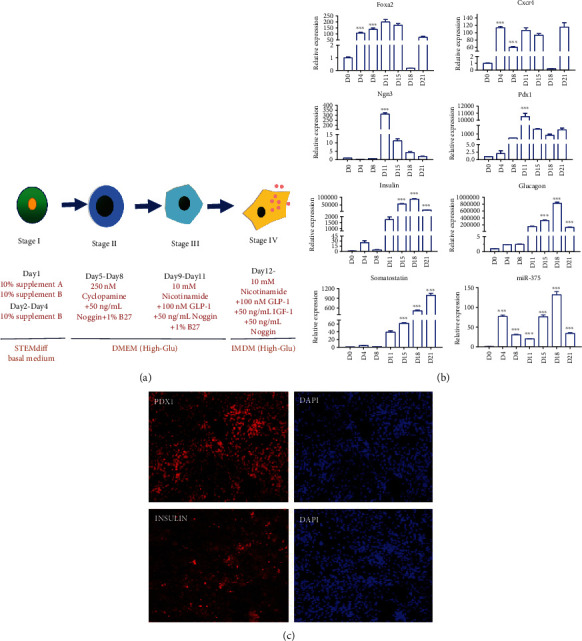
A four-stage protocol for differentiation of human embryonic stem cells to insulin-producing cells. (a) Flowchart of the cell differentiation process. Differentiation is divided into four stages: definitive endoderm (stage I), gastrulation (stage II), endocrine precursor (stage III), and pancreatic endocrine cell (stage IV). (b) The expression of marker genes at each stage of the differentiation process. ∗∗∗ indicates *P* < 0.001, ∗∗ indicates *P* < 0.01, and ∗ indicates *P* < 0.05. (c) Immunofluorescence detection of PDX-1 (samples collected on D13) and INSULIN (samples collected on D21). Scale bar =100 *μ*m.

MEL1 and H9 cells were subjected to the pancreatic differentiation protocol of D'Amour et al. [21, 23], with slight modifications. hESCs were inoculated into Matrigel-coated 24-well plates at a density of 2.1 × 10^5^/cm^2^. The differentiation of cells was directly differentiated by growth factors and small molecules, which mimics in vivo pancreatic organogenesis [23]. The differentiation process comprised four stages: definitive endoderm (stage I), gastrulation (stage II), endocrine precursor (stage III), and pancreatic endocrine cell (stage IV) (see Figure 1(a)). hESCs were differentiated to primitive endoderm using STEMdiff Basal Medium or RPMI as the basic culture medium supplemented with 100 ng/mL activin A and 25 ng/mL Wnt3a.

### 2.2. Transfection of miR-375 Mimics and Inhibitors

The miR-375 mimic and miR-375 inhibitor were purchased from Ambion, TX, USA. The number of miR-375 in miRBase is MIMAT0000728, and the mature miR-375 sequence is UUUGUUCGUUCGGCUCGCGUGA. The Lipofectamine® RNAiMAX Reagent (Invitrogen) was used for transfection.

At the beginning of each stage of differentiation, miR-375 mimics or inhibitors were transfected with liposomes to alter the expression of miR-375, on D0, D3, D6, D10, D13, and D17. At the end of each stage, the cells were collected for analysis. Transfection of the miR-375 mimic/inhibitor was performed in 24-well plates at a concentration of 10 pmol/well with 3 *μ*L of liposomes. The miR-375 mimic transfection samples were labeled as miR-375OE, and miR-375 inhibitor transfection samples were labeled as miR-375IE.

### 2.3. RNA Extraction and Real-Time PCR Analysis

Total RNA was isolated using TRIzol (TransGene Biotech, China) and reverse-transcribed into cDNA using a Reverse Transcription Kit (Takara, Dalian, China). Real-time PCR (RT-PCR) was performed using an ABI Prism 7500 sequence detection system (Applied Biosystems, CA, USA). Each reaction (20 *μ*L) contained 2 *μ*L of cDNA template, 10 *μ*L of SYBR Green Master Mix (Takara, Dalian, China), 0.4 *μ*L of ROX Reference Dye II, 0.8 *μ*L of each of the forward and reverse primers, and 6 *μ*L of RNase-free ddH_2_O. Supplementary Table 1 shows the primer sequences used for RT-PCR. The reactions were performed in a 96-well plate using the following incubation protocol: 95°C for 30 s, followed by 40 cycles of 95°C for 5 s and 60°C for 34 s. The expression of the genes was normalized to the expression of *β*-actin by comparing the cycle threshold (Ct) values. Gene expression was determined by the 2^−ΔΔ^ Ct method and then analyzed for statistical significance.

### 2.4. Sample Collection and Preparation

RNA quantification and qualification of RNA degradation and contamination were performed on 1% agarose gels. The purity of RNA was checked using a NanoPhotometer® spectrophotometer (IMPLEN, CA, USA), and the integrity of RNA was assessed using the RNA Nano 6000 Assay Kit of the Agilent Bioanalyzer 2100 system (Agilent Technologies, CA, USA).

### 2.5. Library Preparation for Transcriptome Sequencing

In total, 1.5 *μ*g RNA per sample was used as the input material for RNA sample preparation. Sequencing libraries were generated using the NEBNext® Ultra™ RNA Library Prep Kit for Illumina® (NEB, USA) in accordance with the manufacturer's recommendations, and index codes were added to attribute sequences to each sample. Briefly, mRNA was purified from total RNA using poly-T-oligo-attached magnetic beads. Fragmentation was conducted using divalent cations at elevated temperature in 5× NEB Next First-Strand Synthesis Reaction Buffer. First-strand cDNA was synthesized using a random hexamer primer and M-MuLV Reverse Transcriptase (RNase H-). Second-strand cDNA synthesis was performed using DNA polymerase I and RNase H. The remaining overhangs were converted into blunt ends via exonuclease/polymerase activities. After adenylation of the 3′ ends of DNA fragments, the NEBNext Adaptor with a hairpin loop structure was ligated to prepare for hybridization. To achieve preferential selection of the cDNA fragments of 250–300 bp in length, the library fragments were purified using the AMPure XP system (Beckman Coulter, Beverly, USA). Then, 3 *μ*L USER enzyme (NEB, USA) was used with size-selected, adaptor-ligated cDNA at 37°C for 15 min followed by 5 min at 95°C before PCR. PCR was then performed using Phusion High-Fidelity DNA polymerase, Universal PCR primer, and Index (X) Primer. Finally, PCR products were purified (AMPure XP system), and library quality was assessed on the Agilent Bioanalyzer 2100 system.

### 2.6. Clustering and Sequencing

Clustering of the index-coded samples was performed at the Novogene Experimental Department (Beijing, China) on a cBot Cluster Generation System using TruSeq PE Cluster Kit v3-cBot-HS (Illumina) in accordance with the manufacturer's instructions. After cluster generation, the library preparations were sequenced on an Illumina Hi-seq platform and paired-end reads were generated.

### 2.7. Prediction of miR-375 Target Genes

The target genes of miR-375 were explored using an online database: TargetScan [27]. TargetScan is a database for predicting miRNA target genes, which mainly searches for conserved 8 mer and 7 mer sites matching each miRNA seed region to predict target genes. One mRNA was considered to be a possible miR-375 target if the interaction was supported in TargetScan. In order to ensure the accuracy of the results, we also use the online database starBase [28] to verify the results and take the intersection of the target genes obtained from the two databases as the final target genes of miR-375.

### 2.8. Data Processing

After the count data were processed by TMM using EdgeR for differential analysis, differentially expressed mRNA was obtained. All *P* values were corrected for the statistical significance of multiple tests using the false discovery rate (FDR). Values of fold change (absolute log2) of ≥1 and FDR adjusted to *P* < 0.05 were considered to be statistically significant, and ClusterProfiler was used for GO (gene ontology) and KEGG (Kyoto Encyclopedia of Genes and Genomes) enrichment analysis of differentially expressed genes. The FPKM (fragments per kilobase per million) information for the differential genes was then converted into TPM information for subsequent analysis.

### 2.9. Time Series Analysis

Short Time-series Expression Miner (STEM) version 1.3.12 was used to perform time series analysis of the relative expression of miR-375 target genes in the miR-375OE group and the control group (using the STEM Clustering Method algorithm, *K* = 10). The relative expression was processed by a logarithmic function. The expression patterns of these genes in different periods were clustered, and the genes were divided into clusters 0 to 9.

### 2.10. Protein Interaction Networks

The STRING database (https://string-db.org/) was used to obtain the protein interaction network for genes related to pancreatic differentiation, and then, Cytoscape 3.7.0 was used to display the PPI (protein-protein interaction) network (Avg). The number of neighbors was 15.738. Larger circles and darker colors indicate a greater degree of connectivity of the gene in the network and a more central role in the network, respectively.

### 2.11. Western Blotting

Sample cells were lysed using a RIPA protein extraction reagent (Beyotime, Beijing, China) supplemented with a protease inhibitor cocktail (Roche, Rotkreuz, Switzerland) and phenylmethylsulfonyl fluoride (Roche, Rotkreuz, Switzerland). A 50 *μ*g sample of the extracted protein was separated via 15% SDS-PAGE, transferred to an Immobilon®-P Transfer membrane (Millipore, USA), and incubated with specific antibodies. The autoradiograms were quantified using densitometry (Quantity One software; Bio-Rad). After the protein content was determined, western blotting was performed. The specific antibodies and dilutions were as follows: anti-PDX1 (1 : 1000) and anti-INSULIN (1 : 800). All antibodies were obtained from Abcam (AB, USA). The target protein levels were normalized to anti-GAPDH (1 : 1000) from Abcam (AB, USA). The secondary antibodies used were horseradish peroxidase-conjugated goat anti-rabbit IgG (1 : 10,000) and goat anti-mouse IgG (1 : 5000) from Abcam (AB, USA).

### 2.12. Statistical Analysis

The Ct value obtained in the qPCR experiment was calculated by the 2^−ΔΔ^ Ct method, and the relative expression of each gene was obtained. Variation between the groups was determined by the analysis of variance. In the analysis of variance, *P* values of >0.05, < 0.05, and <0.01 were considered not significantly different, significantly different, and extremely significantly different, respectively. To reduce the error and obtain statistically significant experimental data, three independent replicates of all experiments in this study were performed.

## 3. Results

### 3.1. hESCs Were Differentiated to IPCs in a Four-Stage Protocol

The four-stage differentiation protocol (see Figure 1(a)) was adopted from previous publications [21, 23]. The expression of *FOXA2*, *CXCR4*, *NGN3*, *PDX1*, *INSULIN*, *GLUCAGON*, and *SOMATOSTATIN* at different stages of differentiation was detected by RT-PCR. *FOXA2* and *CXCR4* were expressed from D4 and maintained throughout all stages of differentiation. The relative expression of *FOXA2* was higher in stage II. The expression of *NGN3* and *PDX1*, markers of endocrine precursor cells, increased from D8, and the relative expression was highest at D11 of differentiation. *INSULIN*, *GLUCAGON*, and *SOMATOSTATIN*, markers of pancreatic endocrine cells, were highly expressed at the end of differentiation. The expression of miR-375 increased after the first stage of differentiation and was maintained at a remarkably high level during differentiation, but fluctuations occurred (see Figure 1(b)). Insulin-like secreting cell differentiation was successfully achieved from H9 and MEL1 hESCs, and *PDX1* and INSULIN were detected by immunofluorescence staining (see Figure 1(c)).

To obtain better differentiation results, we examined the effect of Wnt3a concentration on cell differentiation. First, 0 ng/mL, 25 ng/mL, and 50 ng/mL Wnt3a were added at the beginning of differentiation. There was no significant difference in the morphology of cells in stage I and stage II. When differentiated into stage III, the number of dead cells was significantly higher in the 0 ng/mL Wnt3a group. The cells grown in 25 ng/mL Wnt3a had a lower percentage of dead cells and clear multilayered growth. Compared with the 50 ng/mL and 0 ng/mL Wnt3a groups, the 25 ng/mL group had higher *FOXA2* and *PDX1* expression, as well as higher insulin and glucagon secretion levels; therefore, 25 ng/mL Wnt3a was used for our differentiation protocol (see Figure S1).

### 3.2. miR-375 Participates in the Differentiation of IPC Formation through Different Target Genes at Different Stages

**Figure 2:**
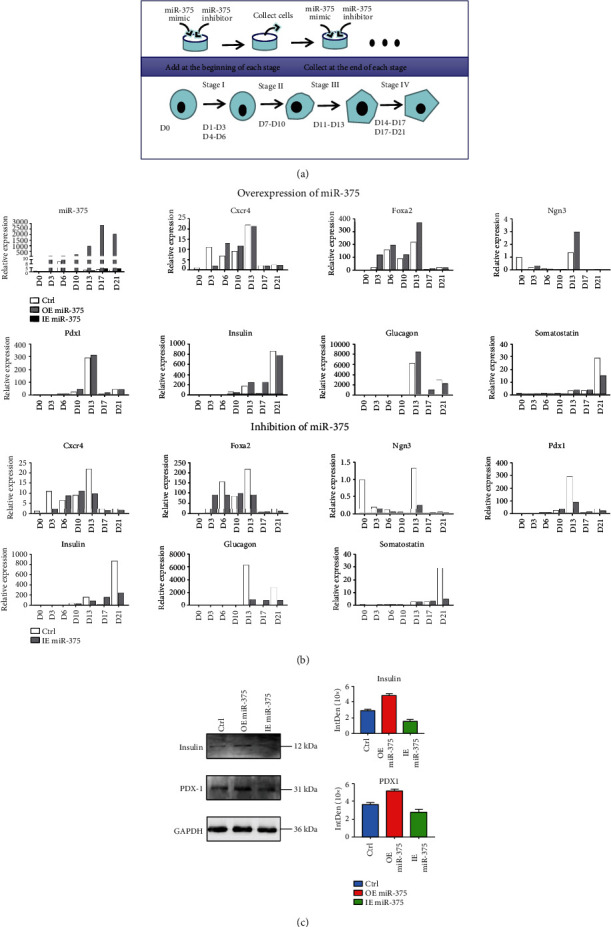
The effects of miR-375 overexpression and inhibition during differentiation of human embryonic stem cells to insulin-producing cells. (a) Illustration of the transfection process. We used liposomes to perform transfection of miR-375 mimics and miR-375 inhibitors at the beginning of each phase (D0, D4, D7, D11, D14, and D18). Samples were collected at the end of each stage (D3, D6, D10, D13, D17, and D21). (b) The effect of the miR-375 mimic and miR-375 inhibition on transcription factors. (c) Western blotting detection and grayscale analysis were used to determine the protein expression of PDX-1 (D13) and INSULIN (D21) after miR-375 overexpression and inhibition.

miR-375 mimics and miR-375 inhibitors were transfected into cells in a 24-well plate for the overexpression (OE) and inhibition (IE) experiments (see Figure 2(a)). The transfection of miR-375 mimics and miR-375 inhibitors did not affect the growth, morphology, and density of cells during differentiation. At the beginning of each stage of differentiation, the cells were transfected; at the end of the stage, the cells were collected and used for gene expression analysis. The expression of miR-375 in cells transfected with miR-375 mimics was significantly higher than that in untransfected cells, whereas its expression in the cells transfected with the miR-375 inhibitor was similar to that in untransfected cells.

RT-PCR was used to detect the relative expression of *FOXA2*, *CXCR4*, *NGN3*, *PDX1*, *INSULIN*, *GLUCAGON*, and *SOMATOSTATIN* after transfection of miR-375 mimics and inhibitors. The transfection of miR-375 mimics at stage I reduced the expression of *CXCR4* but increased *CXCR4* expression at stage II and stage III. The transfection of miR-375 mimics increased the expression of *FOXA2* at all stages and increased the expression of *NGN3* and *PDX1*. The expression of *INSULIN*, *GLUCAGON*, and *SOMATOSTATIN* was increased by miR-375 mimics early in stage IV but was decreased at the end of this stage. The inhibition of miR-375 was achieved by a miR-375 inhibitor. The inhibition of miR-375 increased *CXCR4* expression in stage II and stage III but decreased *CXCR4* expression in the other stages. miR-375 inhibitors also decreased the expression of *FOXA2*, *PDX1*, and *NGN3*, with the exception of a slight upregulation of *FOXA2* in stage I. The expression of *INSULIN*, *GLUCAGON*, and *SOMATOSTATIN* was also downregulated by miR-375 inhibition; however, overall, the effects of miR-375 inhibition were not as clear as the enhancement by the miR-375 mimic (see Figure 2(b)). Western blotting analysis was used to detect the expression of PDX1 protein on D13 and insulin on D21. The results showed that the protein expression of PDX1 and insulin in cells transfected with the miR-375 mimic was higher than that in cells transfected with the miR-375 inhibitor. Therefore, transient overexpression of miR-375 on D10 of differentiation can promote the synthesis of the PDX1 protein, and transient overexpression of miR-375 on D17 of differentiation could promote insulin synthesis on D21 (see Figure 2(c)).

To further investigate how miR-375 influenced pancreatic differentiation, RT-PCR analysis was used to detect changes in other pancreatic-related transcription factors (see Figure S2). The results showed that overexpression of miR-375 caused a decrease in *CAMD1* gene expression at D3, D6, D10, D13, D17, and D21, and *HNF1β* was reduced at D6 and D10. The overexpression of miR-375 in stages II to IV of differentiation reduced the expression of *NOTCH2* (D10, D17, and D21) and *PAX6* (D13 and D17). These results indicated that miR-375 participated in the differentiation of IPCs through different target genes at different stages.

### 3.3. miR-375 Influences Global Gene Expression during Differentiation of hESCs to IPCs

**Figure 3:**
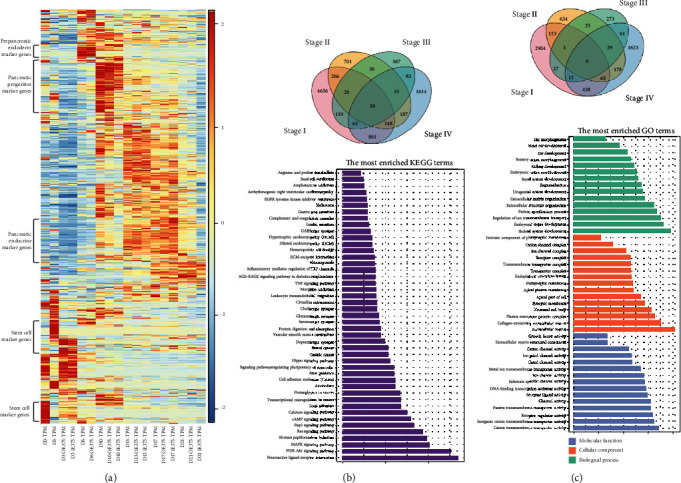
Overall description of differentially expressed genes (DEGs) in the four differentiation stages for control vs. miR-375 overexpression (OE) and control vs. miR-375 inhibition. (a) Heatmap showing the expression of DEGs in the miR-375OE group and the miR-375 inhibition group compared with the control group in different stages of differentiation. Hierarchical clustering was used to cluster gene expression patterns, and its expression level was standardized to TPM. (b) Venn diagram showing the distribution of DEGs in the four stages of differentiation in OE vs. control and bar plot showing the top 45 pathways of KEGG (Kyoto Encyclopedia of Genes and Genomes) enrichment, presented in descending order by gene number. (c) Venn diagram showing the distribution of DEGs in the four stages of differentiation for IE vs. control and bar plot showing the top 15 pathways of three GO (gene ontology) terms: BP (biological process), CC (cellular component), and MF (molecular function).

A comprehensive analysis of RNA-seq data was performed to reveal the changes in the transcriptome during the differentiation of hESCs into pancreatic islet cells. We performed cluster analysis on a total of 21,051 genes in the miR-375OE, miR-375IE, and miR-375 control groups at different stages and annotate potential markers of each stage (see Figure 3(a)). Our results showed that as the differentiation progressed, the overall transcription levels in the cells underwent obvious changes, and stage I, terminal endoderm differentiation, was different from other stages. This suggested that during the process of stem cell differentiation, early differentiation is crucial to the success of differentiation. The expression patterns of the transcriptome are relatively similar in stages II to IV (D13–D17). It is worth noting that the transcription level of cells at the end of differentiation (D17–D21) was changed significantly. Subsequently, the expression of highly expressed genes was significantly reduced. These genes may be related to the expression of *INSULIN* and the differentiation of pancreatic progenitor cells into pancreatic islet-like secretory cells.

The expression of *INSULIN* and *SOMATOSTATIN* in the cells increased significantly on D21, indicating the formation of mature insulin-like secreting cells. To further illustrate the effect of miR-375 on the differentiation process of islet cells, we focused on ~9000 genes. Following overexpression of miR-375, among the 6191 genes that were significantly affected by overexpression of miR-375 in stage I (D0–D6), 4656 genes (approximately 75%) were uniquely affected in this stage. Following inhibition of miR-375, 3591 genes were significantly changed in stage I and 2904 genes (approximately 80%) were uniquely affected at this stage. The proportion of genes that was affected uniquely after miR-375 overexpression was 47.5% in stage II, 47.5% in stage III, and 53.7% in stage IV. The proportion of unique genes affected after miR-375 inhibition was 50% in stage II, 60% in stage III, and 67.2% in stage IV (see Figures 3(b) and 3(c)). These data showed that the genes affected by miR-375 at different stages are clearly different.

Regardless of the overexpression or inhibition of miR-375, more than 40% of the genes affected were unique to that stage. Among these genes, the most significant effect of miR-375 occurred in stage I (D0–D6). We then performed GO function and KEGG pathway enrichment analyses on the differentially expressed genes. GO enrichment analysis showed that the main functions of the enriched mRNA coexpressed with pancreas-related genes were in skeletal system development, collagen-containing extracellular matrix, extracellular matrix, and cation transmembrane transport activity (see Figure 3(b)). KEGG pathway analysis showed that mRNA coexpressed with miR-375 participates in neuroactive ligand interaction in the PI3K-Akt signaling pathway and the MAPK signaling pathway (see Figure 3(c)). Although we did not observe enrichment in the relevant pathways for pancreatic development, our results indicated that miR-375 has a wide range of effects on transcription during the differentiation of ESCs into insulin-producing cells.

### 3.4. miR-375 Target Genes Are Widely Involved in the Differentiation Process

**Figure 4:**
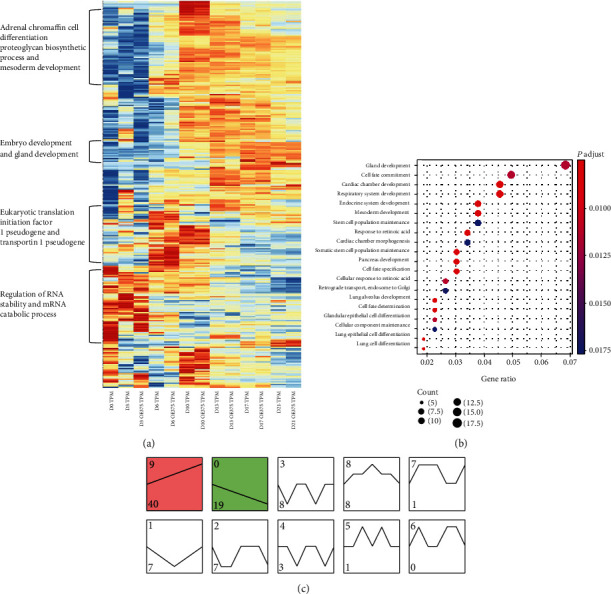
Overall description of 272 possible miR-375 target genes for the four differentiation stages in control vs. OE and control vs. IE. (a) Heatmap showing the expression levels of 272 possible miR-375 target genes in the four differentiation stages in the OE, IE, and control groups. Hierarchical clustering of gene expression patterns was performed, and its expression level was standardized to TPM (transcripts per kilobase of exon model per million mapped reads). (b) Dot plot showing the KEGG enrichment pathways of 272 possible target genes. The abscissa represents the gene enrichment ratio, the bubble size represents the number of enriched genes in this pathway, and the color represents the *q* value (adjusted *P* value). (c) Trend analysis of target gene expression (10 trends) by STEM (Short Time-series Expression Miner). Colored blocks: significantly enriched trend (*P* < 0.05).

To determine the effects of miR-375 on pancreatic differentiation genes, we selected 272 miR-375 target genes to construct a heatmap and categorize these genes (see Figure 4(a)). During stem cell differentiation, the expression patterns of miR-375 target genes were changed significantly. Most of the highly expressed miR-375 target genes were concentrated in stages I to II of differentiation. At the end of differentiation, the expression of miR-375 target genes was universally low. After overexpression of miR-375, most genes were not dramatically altered. Only a small number of genes were significantly increased or decreased after overexpression of miR-375, but this change was often only focused on one or several stages of differentiation.

We also analyzed the GO function enrichment of miR-375 target genes (see Figure 4(b)). We focused on the most enriched gene sets that were related to pancreatic development. In 272 selected miR-375 target genes, genes relating to gland development (20/272) and cell fate commitment (14/272) functions accounted for the largest proportion. Epithelial cell differentiation (6/272) and pancreatic development (9/272) were highly related to pancreatic development. After the GO function enrichment analysis, miR-375 target genes were highly enriched in pancreatic differentiation and cell fate function. These gene sets included transcription factors that are important in the in vitro differentiation of pancreatic cells, such as *HNF1βB*, *PAX6*, *INSM1*, *GATA6*, *PDPK1*, and *INSR*. This result indicated that miR-375 may affect the in vitro pancreatic development of embryonic stem cells through its effects on its target genes.

We also examined the clustering of the expression patterns of miR-375 target genes during the differentiation of control cells, and the genes were divided into clusters 0 to 9. The numbers on the upper left of Figure 4(c) represent clustering, the numbers on the lower left represent the number of genes clustered in different clusters, and the colored modules indicate a significance of *P* < 0.05 (see Figure 4(c)). In our aggregated 10 gene counts, the expression level of gene set 9 (containing 40 genes) continued to increase, and the expression level of gene set 0 (containing 19 genes) continued to decrease. Count 9 includes pancreas development, cell fate commitment, pancreatic *β*-cell differentiation, and other functionally related transcription factors, such as *FOXF1*, *PAX6*, *INSM1*, *GATA6*, *PDPK1*, *NR5A2*, and *INSR*. Count 0 includes gland development, cell fate determination, glandular epithelial cell differentiation, and other functionally related transcription factors, such as *HOXA5*, *LRP5*, *CCDC39*, *KLF4*, *ASCL1*, *RBPJ*, and *JAK2*. Although these genes are the target genes of miR-375 and their functions were related to pancreatic differentiation, cell fate, and other related functions, the expression patterns during differentiation were significantly different. In combination with the other eight sets, it was shown that the target genes of miR-375 follow a variety of expression patterns during pancreatic development and are involved throughout the process of cell differentiation. These results also showed that miR-375 may affect the expression of multiple target genes at different stages of differentiation and that it can affect different targets at the same stage.

### 3.5. Overexpression of miR-375 Leads to Changes in Multiple Signaling Pathways during Pancreatic Development

**Figure 5:**
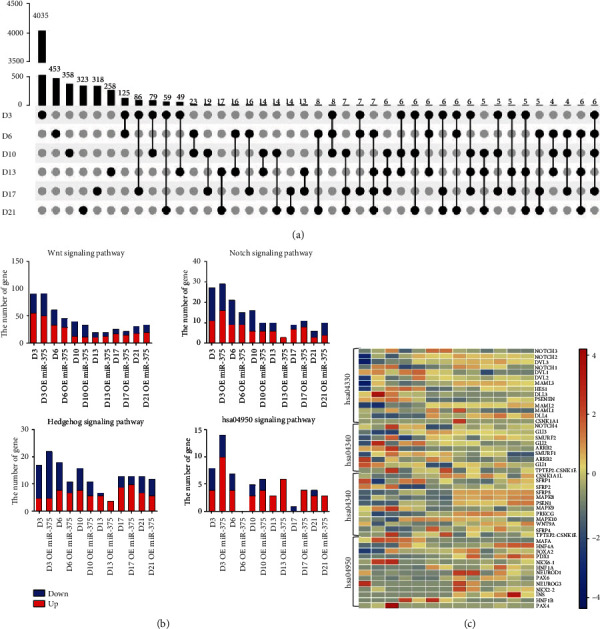
Interaction of genes related to pancreatic differentiation. (a) The number of differential genes obtained by comparing miR-375 with its corresponding control group after overexpression at different stages. (b) The number of genes that overexpressed miR-375 has changed significantly compared with its corresponding control group in different signaling pathways. Blue represents decreased expression, and red represents increased expression. (c) The heatmap shows the expression of important transcription factors in different pathways in the OE group and the control group.

To determine how miR-375 affects pancreatic development, we constructed a map of the unique genes affected at different stages of differentiation (see Figure 5(a)). As the number of unique genes affected in stage I was significantly greater than that in other stages of cell differentiation, there were comparatively more unique gene intersections between D3 and D6, D10, D13, D17, and D21. However, for other stages, no matter whether two or three stages were considered, the number of unique gene intersection was not significantly greater than that at other stages, which indicated that gene expression during the process of pancreatic differentiation of ESCs is clearly stage-specific. This also showed that some unique genes continue to exist and were more clearly expressed. For example, *FRG1* and *KCNK15* were more highly expressed on D10 and D17 but decreased on D13. *DUS4L* was only higher on D10 and D13.

To further study how miR-375 regulated the process of pancreatic differentiation of ESCs, we selected a variety of pathways from early pancreatic development to late insulin secretion-related pathways, such as the WNT signaling pathway (hsa04310) (157 genes), NOTCH signaling pathway (hsa04330) (52 genes), HEDGEHOG signaling pathway (hsa04340) (50 genes), and mature onset diabetes in young people (hsa04950) (including 26 genes) for further analysis. We constructed heatmaps to show the significant changes in genes in these pathways (see Figure 5(b)). Although the differentiation process of hESCs mimics the development of the pancreas in vivo, the influence of the key signaling pathways on cell differentiation is somewhat different to embryonic development. The NOTCH and WNT signaling pathways are mainly enriched in the early stage of islet cell differentiation. The influence of the HEDGEHOG signaling pathway on the differentiation of islet cells is reflected during early and late differentiation. In addition, genes in the mature onset diabetes in young people (hsa04950) pathway (such as *HES1*, *GCK*, *FOXA3*, *MAFA*, *HNF4A*, *HHEX*, and *PDX1*) were increased in stages II to IV; these genes are related to insulin secretion and the maturation of pancreatic *β*-cells (see Figure 5(c)).

To understand the changes in specific genes from each pathway after overexpression of miR-375, we constructed a heatmap from selected genes (see Figure 5(c)). It was found that following the overexpression of miR-375, some genes were increased or decreased significantly. For example, in the early stage of differentiation after overexpression of miR-375, the expression of *NOTCH2* and *DLL3* genes in the NOTCH signaling pathway decreased and that of WNT increased. *CXNK1E* is expressed in the WNT signaling pathway, and *PAX4* is expressed in the hsa04950 pathway. To provide a more accurate illustration of the effects of miR-375 on key genes of pancreatic-related pathways, we used transcriptome data to create a map of the expression levels of key genes after overexpression of miR-375 (see Figure S3). In the NOTCH signaling pathway, the overexpression of miR-375 reduced the expression of *NOTCH2* on D3, D10, D17, and D21 and reduced *MAML1* on D10 and D13. The overexpression of miR-375 on D10 reduced the expression of *DLL4*, and the overexpression of miR-375 after D13 increased the expression of *DLL4*. In the WNT signaling pathway, the overexpression of miR-375 increased the expression of *SMURF1* in the early stages of differentiation (D3–D10). In the HEDGEHOG signaling pathway, the overexpression of miR-375 also increased the expression of *SFRP1* in stage I of differentiation (D3–D10). In the hsa0495 signaling pathway, the overexpression of miR-375 reduced the expression of *HNF1β* and *NEUROG3* in stage II of differentiation. These results indicated that miR-375 is widely affected by pancreatic development-related pathways, and these effects are achieved by altering genes in these pathways.

**Figure 6:**
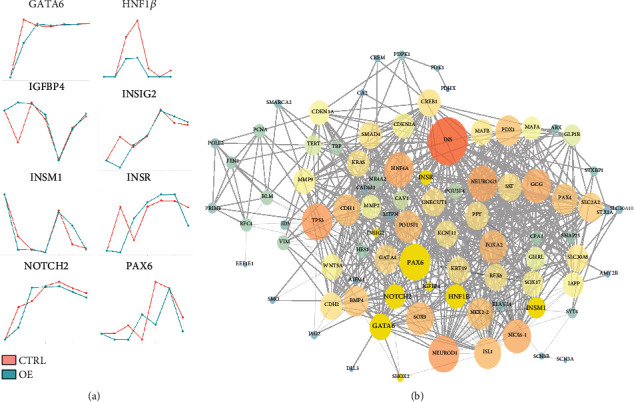
Overexpression of miR-375 can lead to changes in multiple signaling pathways during pancreatic development. (a) The line chart shows the expression level of some differentially expressed genes occupying a central position in protein-protein interaction networks. The red line represents the control group and the green line represents the overexpression group, the abscissa represents the differentiation time, and the ordinate represents the expression of the gene in different differentiation stages (normalized to TPM). (b) The protein-protein interaction network obtained from the STRING database. Each circle represents a gene, and each edge represents a possible interaction between two genes. The size of the circular area represents the importance of the gene in the network. The highlighted genes (black characters on a yellow background) play an important role in the network and need to be analyzed individually and are plotted in (a).

The changes in the target genes after the overexpression of miR-375 as determined RNA-seq data are shown in Figure 6(a). Although the results are different from those obtained by RT-PCR, the changes in expression of the target genes of miR-375 caused by the overexpression of miR-375 were broadly similar. For example, both RT-PCR and RNA-seq data showed that after the overexpression of miR-375, *HNF1β* was reduced on D6, D10, and D21 and *NOTCH2* was reduced on D17 and D21. *GATA6* was decreased on D3, *PAX-6* was decreased on D13, and *ISNM1* was decreased on D17 and D21. The miR-375 target genes *PAX6*, *HNF1β*, *INSM1*, *GATA6*, *NEUROG3*, and pancreatic development-related transcription factors, such as *INS*, *POU5F1*, *GATA4*, and *PDX-1*, occupy important core positions during pancreatic differentiation (see Figure 6(b)); during the differentiation of hESCs into IPCs, miR-375 controls pancreatic-related transcription factors through different target genes, and these target genes vary during each stage.

## 4. Discussion

As one of the most enriched microRNAs during the growth and development of the pancreas, miR-375 is specifically expressed in islet cells and regulates embryonic development of islet; in turn, the process and function of mature pancreatic islet *β*-cells affect the synthesis and secretion of insulin [16]. In mice, miR-375 is highly expressed in islets and is essential for normal glucose homeostasis. Mice lacking miR-375 (375KO) are hyperglycemic, with a greater number of *α*-cells, high plasma glucagon levels, and increased gluconeogenesis and hepatic glucose output [29]. Studies have shown that in human type 2 diabetes and other rodent models with diabetes, the level of extracellular miR-375-3p is affected. Nevertheless, in both humans with type 1 diabetes (T1D) and murine models of T1D, miR-375-3p was consistently found to be upregulated in plasma samples [30]. The circulating level of miR-375 may reflect changes in *β*-cells and may be changed in the blood of patients recently diagnosed with T1D [12, 31]. In short, miR-375 is intricately related to the formation of the pancreas and the secretion of insulin. Studies have confirmed that without the addition of various cytokines and small-molecule compounds, only the overexpression of miR-375 can be used to differentiate iPSCs or ESCs into IPCs after 21 days of in vitro culture, but the differentiation efficiency and maturity are still not satisfactory [32, 33]; after the overexpression of miR-375 in adipose stem cells, induction can also yield more IPCs expressing insulin and Pdx1 [34].

In this study, we transfected liposomes of miR-375 mimics and inhibitors during cell differentiation to overexpress and inhibit miR-375, respectively. RT-PCR, immunofluorescence staining, and western blotting were used to observe the effects of miR-375 on cell differentiation. For 3–4 days of each stage, the high expression of miR-375 can be effectively maintained. However, in the inhibition experiment, the expression of miR-375 was not changed significantly, which may be related to the expression of miR-375. The RNA-seq data showed that the effects of miR-375 were mainly concentrated in endoderm formation (stage I). Both the overall number of affected genes and the extent of gene changes were significantly higher than those in other stages. miR-375 may affect the differentiation of ESCs into endodermal cells. This also indicated that the transformation into endodermal cells was a key step in obtaining insulin-secreting cells.

During stage II to stage IV of differentiation, the number of genes affected by the overexpression of miR-375 was smaller, but the overexpression of miR-375 significantly affects the expression of important pancreatic differentiation-related transcription factors, such as *NGN3* and *PDX1*. This indicates that the number of genes affected by miR-375 is reduced as differentiation progresses, but the impact will be more specific and, further, affect cell fate and insulin secretion through certain transcription factors. Many of the target genes of miR-375 are related to pancreatic development and stem cell fate perception, which confirmed that the means through which miR-375 affects the transformation of ESCs into insulin-secreting cells was closely related to its target genes.

To explain how miR-375 affects the development of the pancreas, we analyzed the effect of miR-375 overexpression on its target genes and pancreatic development-related genes, such as *PDPK1*, *INSM1*, *HNF1B*, *GATA6*, *NOTCH2*, *PAX6*, and *CADM1*. The overexpression of miR-375 reduced the expression of target genes at a specific stage. The expression of the *NGN3* repressor protein, HES1, is restricted by the transmembrane protein *NOTCH* [35]. We speculated that miR-375 reduces the expression of *HES1* by inhibiting the NOTCH-HES1 pathway and further increased the expression of *NGN3*. The increase in *NGN3* expression is more conducive to the differentiation of ESCs into pancreatic islet cells and the secretion of insulin. In addition, the miR-375 target genes *PAX6* and *CADM1* were related to insulin secretion.

It has been reported that miR-375 can promote the silencing of *PAX6* and reduce insulin secretion [36, 37]. *PAX6* inhibits the viability, migration, and invasion of human breast cancer MCF-7 cells, whereas *CADM1* inhibits insulin secretion in INS-1 cells and primary cells [38]. Interestingly, both *PAX6* and *CADM1* are target genes of miR-375 and the overexpression of miR-375 in stages II to III of differentiation reduces the expression of *PAX6* and *CADM1*; however, studies have shown that both *PAX6* and *CADM1* are related to insulin secretion but have the opposite effect. We speculated that during the secretion of hESCs into IPCs, miR-375 simultaneously affects the *CADM1* and *PAX6* genes to alter the level of insulin.

In our experiment, the expression of *INSULIN* increased after miR-375 was overexpressed, which suggested that miR-375 may help to stabilize the level of insulin and that the miR-375 target gene *CADM1* may play a major role in this process. In summary, the expression of miR-375 varied during hESC differentiation and was affected by a miR-375 mimic and a miR-375 inhibitor. miR-375 influences global gene expression and the target genes of miR-375. The overexpression of miR-375 induced changes in multiple signaling pathways during pancreatic development. miR-375 participates extensively in the differentiation of pancreatic *β*-cells through different target genes at different stages. This study provides a basis for further research into how microRNAs affect cell fate and gene transcription.

## Data Availability

The transcriptome sequencing data used to support the findings of this study are available at https://www.ncbi.nlm.nih.gov/bioproject/PRJNA715080.

## Supplementary Materials

Supplementary Materials Supplementary Table 1: primers of RT-PCR. Figure S1: the effects of different concentrations of Wnt3a on cell differentiation and miR-375 expression. Figure S2: the detection of the expression level of pancreatic-related target genes by RT-PCR at different stages after overexpression of miR-375. Figure S3: the effects of overexpression of miR-375 at different stages on the expression level of transcription factors in different signaling pathways.
