# Supplementary material for: miR-375 Promotes Pancreatic Differentiation In Vitro by Affecting Different Target Genes at Different Stages
Source: Stem Cells Int. 2021 Apr 7;2021:6642983. doi: 10.1155/2021/6642983 (PMC8052179; doi:10.1155/2021/6642983)
Supplement: Supplementary Materials — Supplementary Table 1: primers of RT-PCR. Figure S1: the effects of different concentrations of Wnt3a on cell differentiation and miR-375 expression. Figure S2: the detection of the expression level of pancreatic-related target genes by RT-PCR at different stages after overexpression of miR-375. Figure S3: the effects of overexpression of miR-375 at different stages on the expression level of transcription factors in different signaling pathways. [file 6642983.f1.doc]

# miR-375 promotes pancreatic differentiation in vitro by affecting different target genes at different stage

Zhenyu Lu ^1#^, Jing Wang^1#^, Xu Wang^1#^, Zhiying Li^1#^,Dan Niu^1^, Min Wang^1^, Jinzhu Xiang^1^, Yongli Yue^1#*^, Yajuan Xia^2#*^, Xueling Li^1#*^

**Supplementary table 1 Primers of RT-PCR**

| Gene name | Forward primer（5’-3’） | Reverse primer（5’-3’） |
| --- | --- | --- |
| *Gapdh* | TGCACCACCAACTGCTTAGC | GGCATGGACTGTGGTCATGAG |
| *Foxa2* | GGGAGCGGTGAAGATGGA | TCATGTTGCTCACGGAGGAGTA |
| *Cxcr4* | CACCGCATCTGGAGAACCA | GCCCATTTCCTCGGTGTAGTT |
| *Ngn3* | GCTCATCGCTCTCTATTCTTTTGC | GGTTGAGGCGTCATCCTTTCT |
| *Pdx1* | AAGTCTACCAAAGCTCACGCG | GTAGGCGCCGCCTGC |
| *Insulin* | GCAGCCTTTGTGAACCAACA | TTCCCCGCACACTAGGTAGAGA |
| *Glucagon* | AAGCATTTACTTTGTGGCTGGATT | TGATCTGGATTTCTCCTCTGTGTCT |
| *Somatostain* | CCCCAGACTCCGTCAGTTTC | TCCGTCTGGTTGGGTTCAG |
| U6 | ACGATACAGAGAAGATTAGCATGG | GATCGCCCTTCTACGTCGTAT |
| miR-375 | TATTCGTTCGGCTCGCGTG | GATCGCCCTTCTACGTCGTAT |
| HNF1β | ACCAAGCCGGTCTTCCATACT | GGTGTGTCATAGTCGTCGCC |
| GATA6 | CAGTTCCTACGCTTCGCATC | TTGGTCGAGGTCAGTGAACA |
| INSM1 | GTCCACGCCCGTTTCCTACC | CCAGGTTGAAGCTGCGTTC |
| NOTCH2 | CAACCGCAATGGAGGCTATG | GCGAAGGCACAATCATCAATGTT |
| PAX6 | TGGGCAGGTATTACGAGACTG | ACTCCCGCTTATACTGGGCTA |
| CADM1 | ATGGCGAGTGTAGTGCTGC | GATCACTGTCACGTCTTTCGT |
| PDPK1 | GGAACAGCGCAGTACGTTTCT | CTCGTTTCCAGCTCGGAATGG |


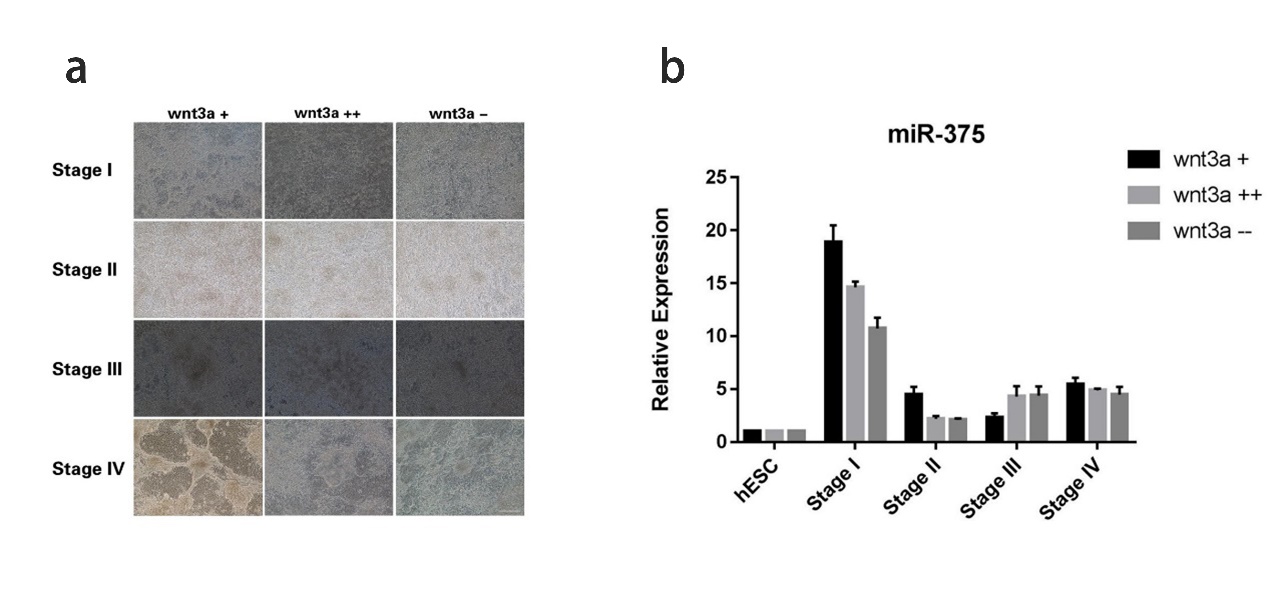


**Figure S1 The effects of different concentration of Wnt3a on cell differentiation and miR-375 expression.** A. Stage I, Stage II, Stage III and Stage IV indicate stages of differentiation, wnt3a + ,wnt3a ++ and wnt3a ̶ indicate the medium of the first protocol of adding 25 ng/mL wnt3a, 50 ng/mL wnt3a and without wnt3a. (The scale is 200 μm). B. The effect of overexpression of MIR-375 at different stages on the expression levels of important transcription factors in different signaling pathways.


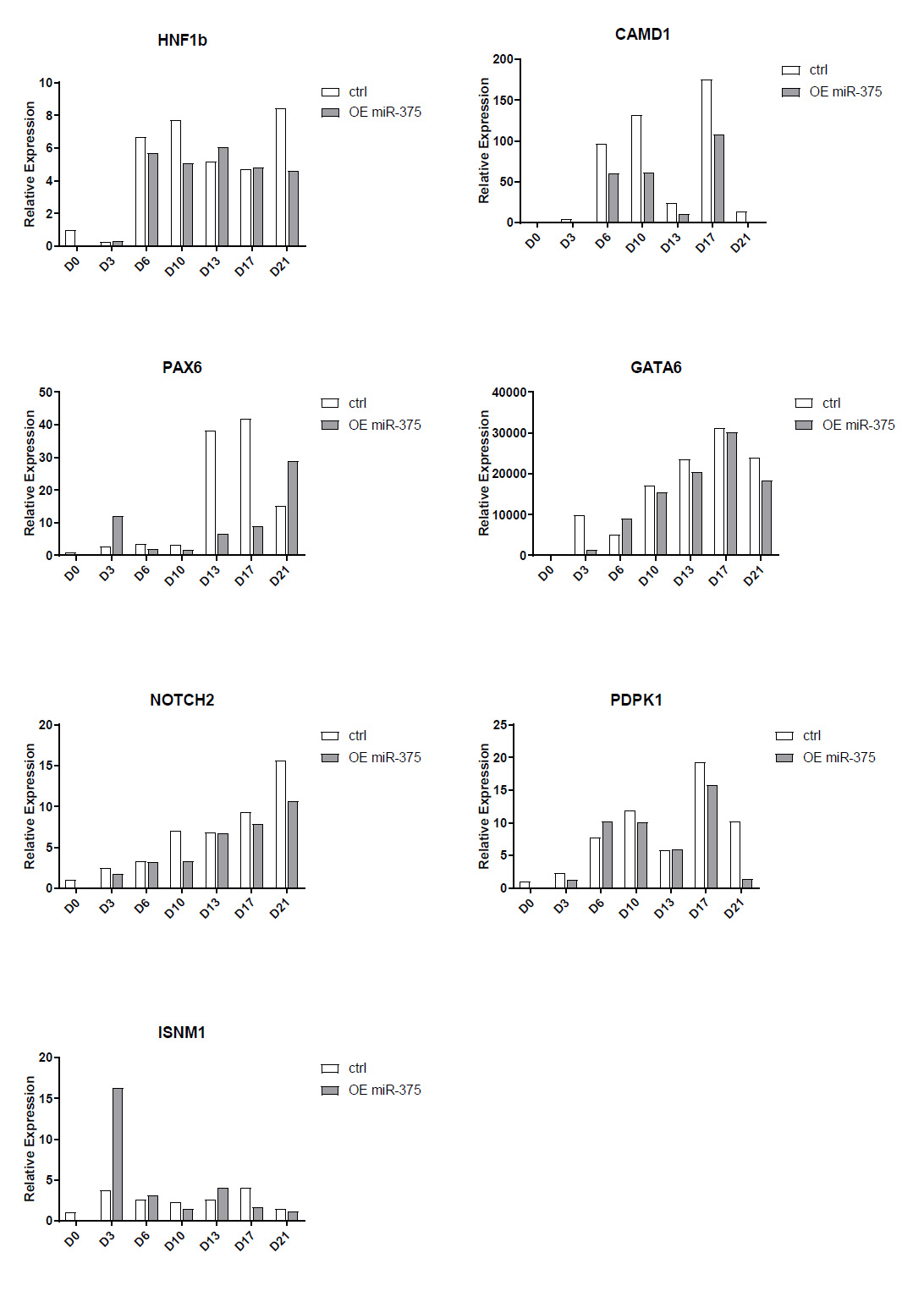


**Figure S2 The detection of the expression level of pancreatic-related target genes by RT-PCR at different stages after overexpression of miR-375.**


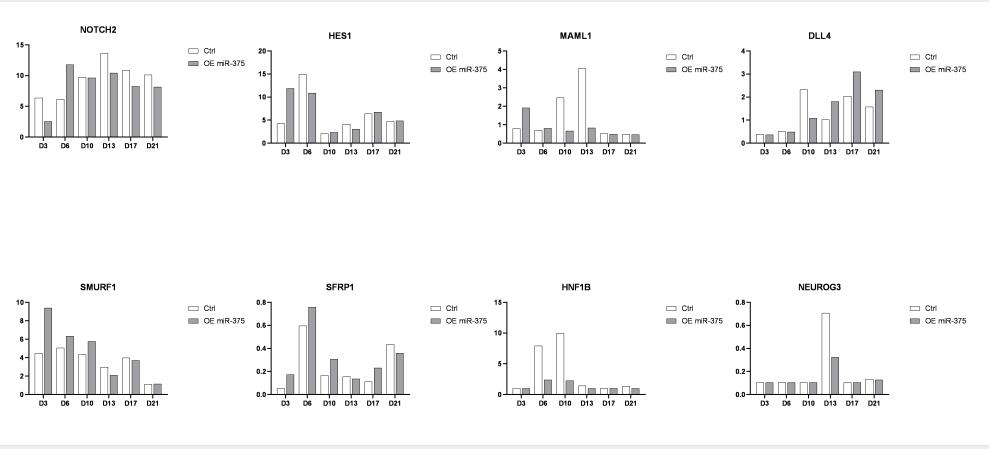


**Figure S3 The effects of overexpression of miR-375 at different stages on the expression level of transcription factors in different signaling pathways.**
